# Trends in clinical and basic research on rotator cuff tears in Japan

**DOI:** 10.1007/s00264-026-06945-6

**Published:** 2026-07-06

**Authors:** Nobuyuki Yamamoto, Eiji Itoi

**Affiliations:** 1 Tohoku University School of Medicine, Sendai, Japan; 2 Tohoku Rosai Hospital, Sendai, Japan

**Keywords:** Rotator cuff tear, Clinical research, Basic research, Trends in Japan

## Abstract

**Introduction:**

Research on rotator cuff tears in Japan was initially characterized by studies clarifying prevalence, asymptomatic tears, and natural history. It subsequently developed toward evaluation of outcomes after arthroscopic repair, postoperative tendon integrity, and long-term results of conservative treatment.

**Methods:**

This article reviews clinical and basic research on rotator cuff tears that has been conducted in Japan.

**Results:**

Representative examples include arthroscopic superior capsule reconstruction (SCR), rotator cuff reconstruction combined with small-head hemiarthroplasty, arthroscopic muscle advancement, and infraspinatus rotational transfer. More recently, basic research on rotator cuff tears in Japan has shifted its focus from simply how to repair the torn tendon to how closely the tendon-bone insertion can be regenerated toward its native structure, and what biological and mechanical conditions can reduce retear.

**Discussion & Conclusion:**

Because reverse total shoulder arthroplasty (rTSA) was introduced in Japan in 2014, later than in many other countries, several Japan-specific surgical techniques for massive rotator cuff tears were developed during the preceding period.

## Epidemiologic research: Important contributions from Japan

Among Japanese studies on rotator cuff tears, community-based epidemiologic research has had a particularly large international impact. Yamamoto et al. [1] investigated the prevalence and risk factors for rotator cuff tears in the general population, including both symptomatic and asymptomatic tears, and showed that age, dominant arm, and history of trauma were associated with rotator cuff tears. In another community screening study, Minagawa et al.[2] reported that the prevalence of full-thickness rotator cuff tears in the general population was 22.1%, increased with age, and that asymptomatic tears were approximately twice as common as symptomatic tears. Both studies used ultrasonography, which has been widely adopted in Japan since the 1990 s, and may therefore be regarded as research made possible by the widespread use of ultrasound in Japan.

## Pathophysiologic research: Clarifying tear progression

Community screening studies demonstrated that the mere presence of a tear does not necessarily correspond to symptoms or functional impairment. As a result, important current research questions include which tears progress, which tears become symptomatic, and why function is preserved in some shoulders. Because MRI is relatively inexpensive in Japan compared with other countries, many MRI-based studies have also been conducted. Yamamoto et al.[3] prospectively investigated, using MRI, how frequently symptomatic rotator cuff tears progress during conservative follow-up and what factors are associated with tear progression. They reported that symptomatic rotator cuff tears progressed in approximately half of cases, and that the risk factors for progression were medium-sized tears, full-thickness tears, and smoking.

## Conservative treatment: Evaluation of long-term outcomes

Important reports on the long-term results of conservative treatment have also come from Japan. Kijima et al.[4] followed patients with rotator cuff tears treated conservatively and reported that, 13 years after diagnosis, approximately 90% of shoulders had no pain or only mild pain, and approximately 70% had no difficulty with activities of daily living. This study is important because it showed that not all rotator cuff tears require immediate surgery, and that conservative treatment can be an effective option, especially in older or low-demand patients. At the same time, the study also suggested that younger patients tended to have persistent long-term pain and disability in activities of daily living, indicating the need to select treatment based on age, activity level, and tear size.

## Surgical treatment: Anchorless arthroscopic rotator cuff repair

Since the 2000 s, arthroscopic rotator cuff repair has become widely used in Japan. Surgical techniques have evolved from mini-open to single-row repair, double-row repair, suture bridge repair, and transosseous-equivalent repair. Within this context, anchorless rotator cuff repair represents a distinctive surgical technique seen in Japan [5]. This technique mainly consists of arthroscopic transosseous suture repair, in which transosseous suturing is performed arthroscopically using a K-wire with a hole at the tip. Reported advantages of anchorless repair include easier revision surgery after retear, avoidance of anchor pullout and implant-related complications, and lower implant cost.

## Japan-specific surgical procedures for primarily irreparable rotator cuff tears

Traditionally, treatment options for massive or irreparable rotator cuff tears have included partial repair, debridement, tendon transfer, hemiarthroplasty, and rTSA. However, in relatively young patients with only mild arthritic change, treatment strategies that improve shoulder function while avoiding arthroplasty have been sought. One procedure that originated in Japan and has spread internationally is SCR. Mihata et al.[6–10] reported the clinical results of arthroscopic SCR for irreparable rotator cuff tears, and the procedure attracted international attention as a joint-preserving operation that can improve shoulder function and relieve pain. Subsequent studies have evaluated long-term outcomes beyond 10 years, graft healing, retear, return to sports, and the limits of indications. A long-term follow-up report also indicated that SCR using a fascia lata autograft provides functional improvement and pain relief for irreparable rotator cuff tears [11].

Rotator cuff reconstruction combined with small-head hemiarthroplasty, developed and refined by Suenaga and colleagues [12], is a procedure that facilitates reconstruction of the rotator cuff and transferred tendons by reducing the size of the humeral head. Through medialization of the center of rotation, the procedure aims to restore function in patients with irreparable rotator cuff tears and cuff tear arthropathy. Long-term follow-up studies have reported that clinical improvement can be maintained for more than ten years.

Infraspinatus rotational transfer has also been reported. In this procedure, the remaining infraspinatus is rotated anterosuperiorly to reconstruct the rotator cuff defect. Ishitani et al. [13] evaluated two year outcomes after infraspinatus transfer in 19 patients with massive rotator cuff tears. The retear rate was 5.3%, and the JOA score improved from six months postoperatively. The authors considered infraspinatus transfer to be a useful procedure with a low retear rate, particularly for improving elevation function. A characteristic feature of this technique is that it uses a local rotator cuff muscle rather than a distant tendon transfer such as the latissimus dorsi, making it easier to preserve the anatomic continuity of the rotator cuff.

Muscle advancement, or the Debeyre-Patte procedure [14], is a technique for large and massive rotator cuff tears that are difficult to repair primarily. By advancing the supraspinatus and infraspinatus muscle–tendon units laterally, the torn cuff edge can be brought to the greater tuberosity, allowing anatomic repair at the tendon-bone junction. In Japan, this procedure has also been performed arthroscopically [15, 16]. Unlike patch procedures or tendon transfers, its key feature is that it aims for primary repair using the native rotator cuff.

Extreme medialization is an arthroscopic procedure for large and massive rotator cuff tears that are difficult to repair [17]. By resecting the superior aspect of the humeral head and shifting the cuff insertion markedly medial to the top of the humeral head, the native rotator cuff can be repaired under low tension. Unlike SCR, patch procedures, or tendon transfers, this method can be performed as an extension of arthroscopic rotator cuff repair without using a graft. Short-term results have shown favorable clinical improvement and a low retear rate. However, further study is needed regarding changes in moment arm caused by excessive medialization and possible long-term joint changes.

rTSA was approved in Japan in April 2014 and clinical use began. Given the high complication rates during the early introduction period in other countries, and from the perspective of overcoming device lag in Japan, rTSA was introduced without conducting clinical trials. Therefore, complete case registration and guideline compliance are required for rTSA use. The guidelines mandate attendance at training courses based on strict surgeon qualification criteria, which ensures acquisition of the necessary knowledge and skills regarding rTSA. Under the current guidelines, the introduction of rTSA in Japan has been achieved more smoothly than expected. The annual number of procedures was approximately 600 cases in 2014 at the time of introduction, but this number has increased year by year, reaching 5,844 cases in 2023, nearly 10 times the initial number in 2014.

## Basic research: Tendon-bone healing, enthesis regeneration, cells, and biomaterials

Basic research on rotator cuff tears in Japan has shifted its emphasis from how to repair the torn tendon to how closely the tendon-bone insertion can be regenerated toward its native structure, and what biological and mechanical conditions can reduce retear.

### Tendon-bone healing and enthesis regeneration

This is one of the central themes of current research. Studies have examined whether the native enthesis can regenerate after rotator cuff repair, and whether the repaired tendon matures histologically and approaches normal mechanical strength. Ide et al. [18] showed that even 12 months after patch grafting, the native enthesis was not completely regenerated and mechanical strength remained inferior to normal. Although the repaired site resembled a normal structure macroscopically, histologically it remained in an incomplete healing state. Biomechanical strength reached only approximately 48% of normal, raising concerns about long-term structural vulnerability. The authors concluded that although patch grafting is a useful option for reducing retear after large and massive tears, it does not achieve complete tendon-bone healing, and further optimization of tissue-engineering approaches and postoperative rehabilitation protocols is needed.

### Biologic augmentation to promote tendon-bone healing

Several studies have investigated whether bFGF, hyaluronic acid, corticosteroids, platelet-rich plasma, cell therapy, scaffolds, and other approaches can improve healing of the rotator cuff tendon and the tendon-bone interface. Tokunaga et al. [19] reported the effects of bFGF on rotator cuff healing. Their study clearly showed that FGF-2 stimulates proliferation and tenogenic differentiation of tendon progenitor cells, improves rotator cuff healing through generation of tenomodulin-positive tenocytes, and provides a scientific basis for regenerative medicine strategies for rotator cuff injury. Nakamura et al. [20] examined the effects of corticosteroids and hyaluronic acid on torn tendons in vitro and in a rat model. They reported that corticosteroid injection for rotator cuff tears delays tendon healing and decreases early postoperative biomechanical strength by inducing apoptosis and suppressing proliferation of tendon fibroblasts. In contrast, hyaluronic acid did not adversely affect tendon fibroblasts or inhibit tendon healing, suggesting that it may be a safer alternative.

### Footprint preparation and the bone-bed environment

Research has also examined how the footprint should be prepared during rotator cuff repair to improve tendon-bone healing. In a rat rotator cuff repair model, Nakagawa and colleagues [21] investigated the histologic and biomechanical effects of footprint preparation on tendon-bone healing. Their findings suggest that cancellous bone should not be excessively exposed during medialized rotator cuff repair. Excessive removal of bone structure may impair tendon-bone healing. The study highlights the importance of balancing appropriate marrow stimulation with preservation of the native structure in footprint preparation, and provides a scientific rationale for optimizing biological healing after rotator cuff repair.

### Repair tension and blood flow

An important recent topic is the observation that excessively high repair tension can reduce microvascular blood flow within the rotator cuff and thereby adversely affect healing. Miyake et al. [22] reported that excessive repair tension decreases microvascular blood flow within the rotator cuff. This was the first study to directly and simultaneously measure repair tension and blood flow at five sites using laser Doppler flowmetry.

### Fatty change and muscle atrophy of the rotator cuff muscles

Muscle atrophy and fatty change are major subjects of research as irreversible changes after rotator cuff tears. Using a mouse model, investigators have examined how time after tear and aging influence fatty change [23]. These studies indicate that aging is critically involved in the pathophysiology of fatty change and fibrosis after rotator cuff tears, and that muscle degeneration progresses over time in aged mice. This suggests that aging itself is an independent risk factor for muscle degeneration.

## Data Availability

No datasets were generated or analysed during the current study.
